# The Global Dilemma of Soil Legacy Phosphorus and Its Improvement Strategies under Recent Changes in Agro-Ecosystem Sustainability

**DOI:** 10.1021/acsomega.3c00823

**Published:** 2023-06-16

**Authors:** Farheen Solangi, Xingye Zhu, Shumaila Khan, Nazia Rais, Asma Majeed, Muhammad Azeem Sabir, Rashid Iqbal, Shehzad Ali, Aqsa Hafeez, Baber Ali, Sezai Ercisli, Ehlinaz Torun Kayabasi

**Affiliations:** † Research Centre of Fluid Machinery Engineering and Technology, Jiangsu University, Zhenjiang, Jiangsu 212013, China; ‡ Khwaja Fareed University of Engineering & Information Technology, Rahim Yar Khan, Punjab 64200, Pakistan; § Department of Soil Science, Sindh Agriculture University, Tandojam, Sindh 70060, Pakistan; ∥ Institute of Agro-Industry and Environment, The Islamia University of Bahawalpur Pakistan, Bahawalpur, Punjab 63100, Pakistan; ⊥ Institute of Forest Sciences, Faculty of Agriculture and Environment, The Islamia University of Bahawalpur, Bahawalpur, Punjab 63100, Pakistan; # Department of Agronomy, Faculty of Agriculture and Environment, The Islamia University of Bahawalpur Pakistan, Bahawalpur, Punjab 63100, Pakistan; 7 Department of Environmental Sciences, Quaid-i-Azam University, Islamabad 45320, Pakistan; 8 Department of Plant Sciences, Quaid-i-Azam University, Islamabad 45320, Pakistan; 9 Department of Horticulture, Faculty of Agriculture, Ataturk University, 25240 Erzurum, Türkiye; 10 HGF Agro, Ata Teknokent, TR-25240 Erzurum, Türkiye; 11 Department of Agricultural Economy, Faculty of Agriculture, Kocaeli University, 41285 Kartepe, Türkiye

## Abstract

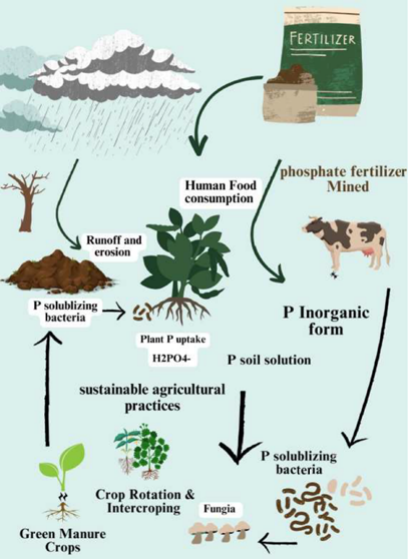

Phosphorus (P) is one of the six key elements in plant nutrition and effectively plays a vital role in all major metabolic activities. It is an essential nutrient for plants linked to human food production. Although abundantly present in both organic and inorganic forms in soil, more than 40% of cultivated soils are commonly deficient in P concentration. Then, the P inadequacy is a challenge to a sustainable farming system to improve the food production for an increasing population. It is expected that the whole world population will rise to 9 billion by 2050 and, therefore, it is necessary at the same time for agricultural strategies broadly to expand food production up to 80% to 90% by handling the global dilemma which has affected the environment by climatic changes. Furthermore, the phosphate rock annually produced about 5 million metric tons of phosphate fertilizers per year. About 9.5 Mt of phosphorus enters human food through crops and animals such as milk, egg, meat, and fish and is then utilized, and 3.5 Mt P is physically consumed by the human population. Various new techniques and current agricultural practices are said to be improving P-deficient environments, which might help meet the food requirements of an increasing population. However, 4.4% and 3.4% of the dry biomass of wheat and chickpea, respectively, were increased under intercropping practices, which was higher than that in the monocropping system. A wide range of studies showed that green manure crops, especially legumes, improve the soil-available P content of the soil. It is noted that inoculation of arbuscular mycorrhizal fungi could decrease the recommended phosphate fertilizer rate nearly 80%. Agricultural management techniques to improve soil legacy P use by crops include maintaining soil pH by liming, crop rotation, intercropping, planting cover crops, and the consumption of modern fertilizers, in addition to the use of more efficient crop varieties and inoculation with P-solubilizing microorganisms. Therefore, exploring the residual phosphorus in the soil is imperative to reduce the demand for industrial fertilizers while promoting long-term sustainability on a global scale.

## 1 Introduction

Phosphorus (P) is unique among all the macronutrients and essential for crops.^1,2^ Soil P plays a key role in various processes, i.e., energy transformation, enzyme activation, photosynthesis, formation of nucleic acid, and adenosine triphosphate (ATP) synthesis.^3^ The addition of P fertilizer increases the amount of phosphorus in the soil, mainly known as accumulated (legacy) phosphorus, which is not immediately available for plant uptake.^4^ The legacy P potentially plays a key role in sustaining crop production, with lower P input supplies and reduced P transfers from land to water if crops can efficiently access this P.^5^ The legacy P is commonly found in two different forms, e.g., organic and inorganic. Both different forms of P depend on soil mineralogy. Long-term buildup of P in the soil is undesirable in agricultural farming systems. However, one important economic and environmental problem related to the development and intensification of agricultural land is the substantial increase in fertilizer demand to sustain crop production.^6^

Presently, there are different challenges related to obtaining legacy soil P as a resource for modern agriculture. Based on previous studies, organic P may constitute a minor portion of legacy P. Moreover, it is noted that non-fertilizer-derived soil organic P may contribute less to plant uptake than inorganic P in soils highly enriched with inorganic legacy P.^7^ The accumulated P naturally becomes obtainable for plant uptake. However, some management strategies can be used to reduce P losses while improving profitability opportunities, which are briefly discussed below in the Review. Phosphate use management at the farm level aims to reduce phosphate use and prevent excess phosphorus from entering the environment. This goal can be achieved by growing crops such as green manure crops that require a small quantity of P fertilizer. These crops can efficiently uptake P and then are mostly utilized for farming practices to increase nutrient levels in the soil. Additionally, other sustainable agricultural practices, including crop rotation, intercropping, exploitation of P-solubilized microorganisms, and tillage practices, dilute the improved topsoil P content through the plough layer.^8^ However, these methods are not easily adopted for wide use because a high amount of fertilizer spoils the surface and groundwater quality, while tillage stimulates mineralization and increases the risk of soil erosion.

Improving soil legacy P availability for plant uptake requires a detailed understanding of the occurrence and reactivity of various soil chemical P forms, which are described in section 1.1 of this Review. Several studies have focused on root physiology and morphological mechanisms, and there is currently limited knowledge about P diagnostic techniques for different crops and soil phosphorus loss and its management.^9−11^ The selected objectives of this Review are (1) to understand the bioavailability of various chemical forms of P in the soil as well as how both forms potentially interact with soil properties, such as soil pH, organic matter content, and other soil properties; (2) to understand the legacy P changes with organic and inorganic fertilizer applications in relation to their potential bioaccessibility; and (3) the diagnosis of P deficiency techniques and challenges for obtaining P from soil that can be used for various crop production. We highlight how plant-based strategies can be useful for sustainable agriculture and the challenges faced by current methods of obtaining native soil phosphorus, such as phosphorus-dissolving microorganisms, phosphorus hydrolases, incorporation of different crop residues, and green manure practices.

### 1.1 Legacy P Fractions

**Figure 1:**
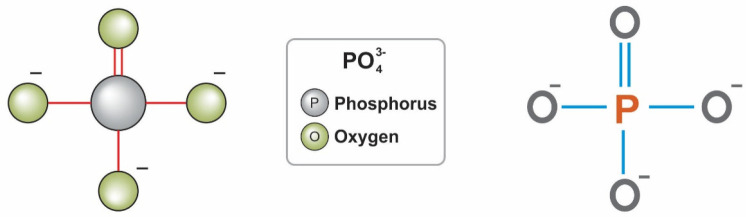
Presented phosphate structure.

**Figure 2:**
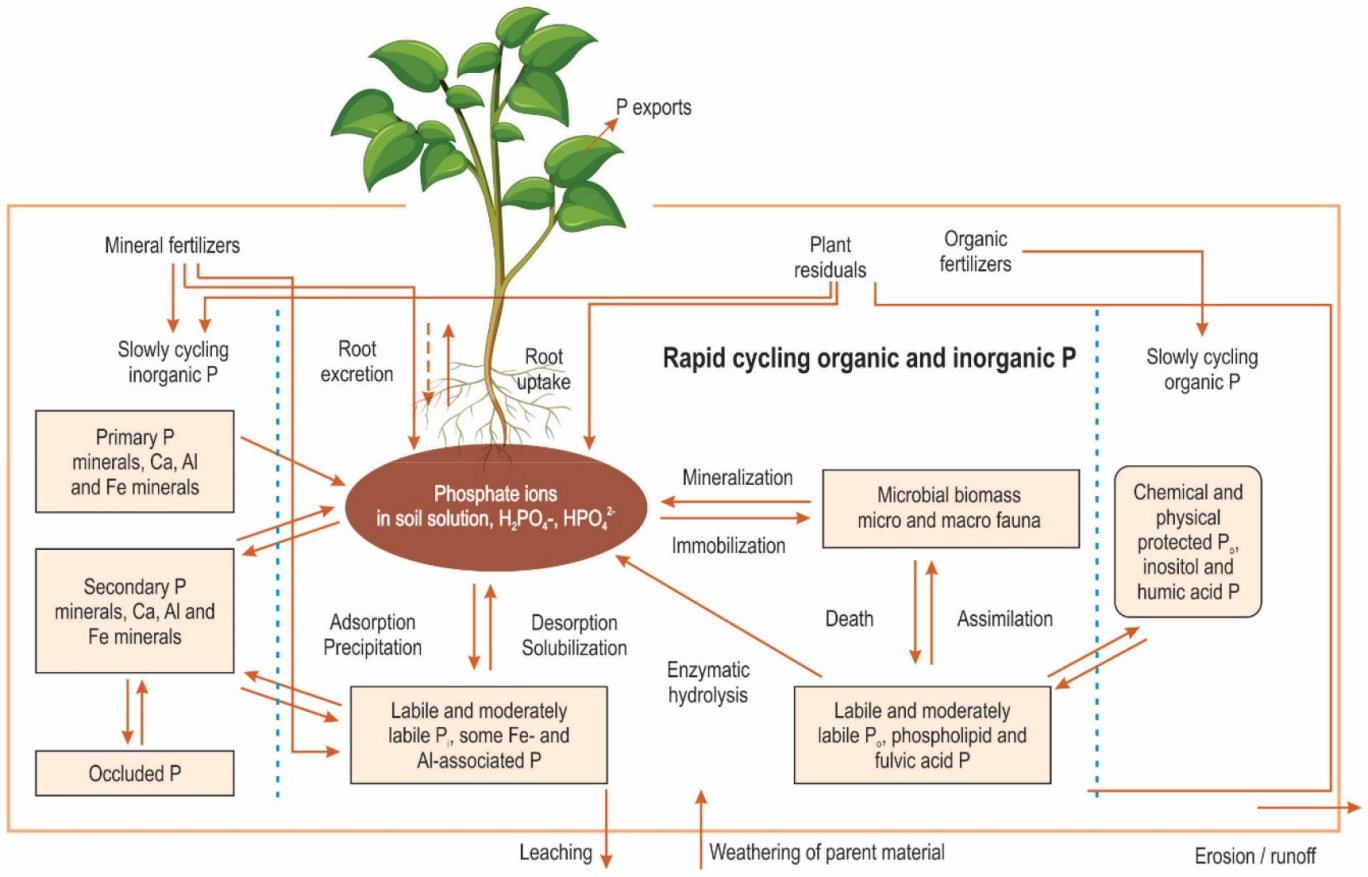
Diagram showing the chemical and biological conversion of P in soil solution. Modified from refs (21) and (22).

Besides all the facts, in most soil conditions, P is the least mobile macronutrient; a steady and adequate supply of P is required for root growth and seed production and is necessary for the growth of the chemical, physiological, and biochemical functions of plants. If phosphorus found in a labile pool (available) has weak adsorption with oxides, a semilabile pool depends on strong adsorption with these oxides and is precipitated with aluminum, iron, and calcium found in unavailable forms (nonlabile P).^12^ Plants can easily uptake soil organic P in the orthophosphate (PO_4_^3–^) form, usually monovalent and dihydrogen phosphate ions shown in Figure 1. Orthophosphate (Pi) is a major regulator of carbon metabolism in plants. Total phosphorus is found in the natural environment at levels from 10% to 90%, and a large proportion of total phosphorus in soil (about 30–65%) is usually in the inorganic form. The P cycle is shown in Figure 2; there are many biotic and abiotic reactions are related to the P cycle in the soil, some occurring within a few seconds and others taking time over many years.^13^ The early breakdown can often be the rate-limiting step for organic P mineralization.^14^ The adsorption/desorption reaction controls labile P in the solid phase, which is related to positively charged minerals Fe and Al, and specific adsorption occurs when P ions are exchanged on the surface of hydroxyl and Al and Fe oxides and hydrated oxides.^15,16^

Organic phosphorus only becomes available after mineralization by a specific phosphatase-catalyzed biodegradation process from inorganic phosphorus, where solubility equals bioavailability. The most common classes of organic P are orthophosphate monoesters and diesters, where monoesters typically comprise 50–70% of organic soil P.^17^ P deficiency affects the balance between the synthesis and catabolism of carbon metabolites. P deficiency usually occurs in the soils and does not move readily to roots through mass flow and diffusion like nitrate ions. Therefore, plants absorb phosphorus from the soil in three different processes: mass flow, diffusion, and interception exchange the major portion. Only a tiny (4%) amount of phosphate ions is delivered to the plant roots via mass flow, and a major portion is exchanged by a diffusion process.^18,19^ It has been recognized that phosphorus availability and its deficiencies may depend on soil characteristics and the labile P portion. Globally, about 5.7 billion hectares of land has low available P for sustaining the production of plants.^20^

### 1.2 Soil pH and Phosphorus Availability

Soil pH (master variable) mainly plays an essential role in soil chemistry to promote nutrient availability and mobility. Globally, around 40% of acid-weathered soils are P deficient due to fixations of insoluble complexes with cations such as Al and Fe in the tropical and subtropical regions.^23^ In contrast, alkaline-weathered soil contains a high amount of Ca^2+^ and Mg in soil solution commonly found in semiarid and arid climates, limiting P solubility by soluble Ca–P compounds. However, maximum P availability for plants is mostly found in neutral soil pH values.

**Figure 3:**
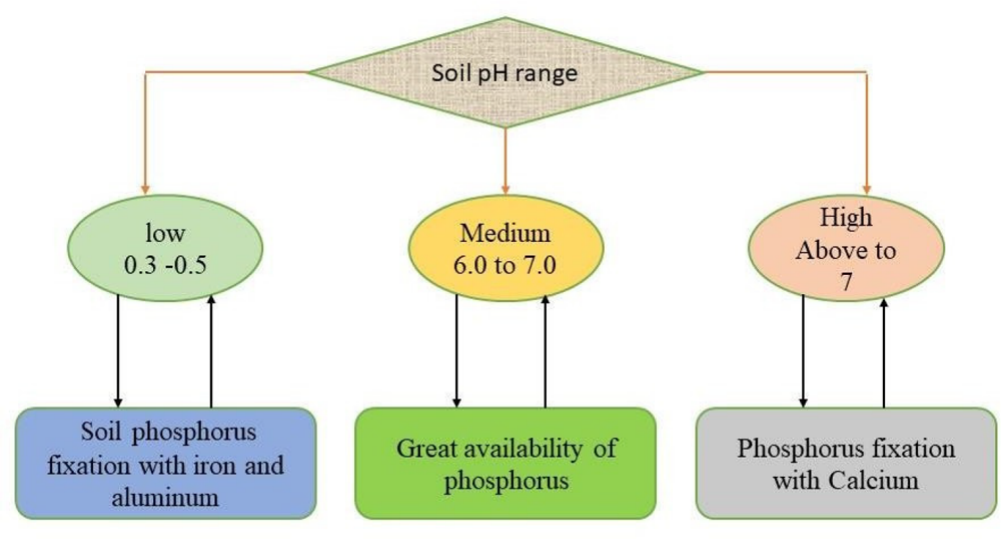
Relationship between soil pH and phosphorus availability.

Therefore, it is recommended to check and optimize soil pH for the availability of nutrients to plants. Lime application may be effective in enhancing soil pH instead of applying fertilizer, which ultimately leads to environmental degradation. For example, Figure 3 shows the link between the pH range and the availability of phosphorus in the soil. Earlier research indicates that the rate of lower P uptake in barley and maize crop is noted at near to neutral pH in acid pH.^24^ It is considered that some species of plants prefer the acidic pH environment and can uptake P at higher rates in the low pH range for their yield. Furthermore, an analysis of P uptake and soil pH might describe the condition of the soil and its solution. In other words, pH conditions might be changed depending on P availability in hydroponic conditions and soil status. Previous analysis showed the pH was mainly changed in the rhizosphere and bulk soil in pea and wheat plants. The pH in rhizosphere soil was only one unit lower compared with that of bulk soil. If the soil can provide sufficient phosphorus in the solution, which plants potentially uptake by roots, one must be careful in assigning alterations obtained for P and plant biomass that occur with pH adjustments to P availability. Minimum P uptake was recorded in barley and maize grown in soil-less solution at a neutral level of pH.^24^ It is considered that some species of plants prefer the acidic pH environment and can uptake P at higher rates for their yield at a low pH range. For example, grasses can solubilize the P that is normally under an acidic environment, where P is rarely formed due to aluminum and iron minerals.^25^ Additionally, the pH, which dominates P release mechanisms, might be changed quickly and improve P solubility. Generally, some studies indicate that increasing the pH range increases P availability in noncalcareous soil.^26^ This may happen due to stability among whole P release mechanisms, which could vary between soils.

## 2 P Deficiencies and Diagnosis Techniques in Various Crops

**Figure 4:**
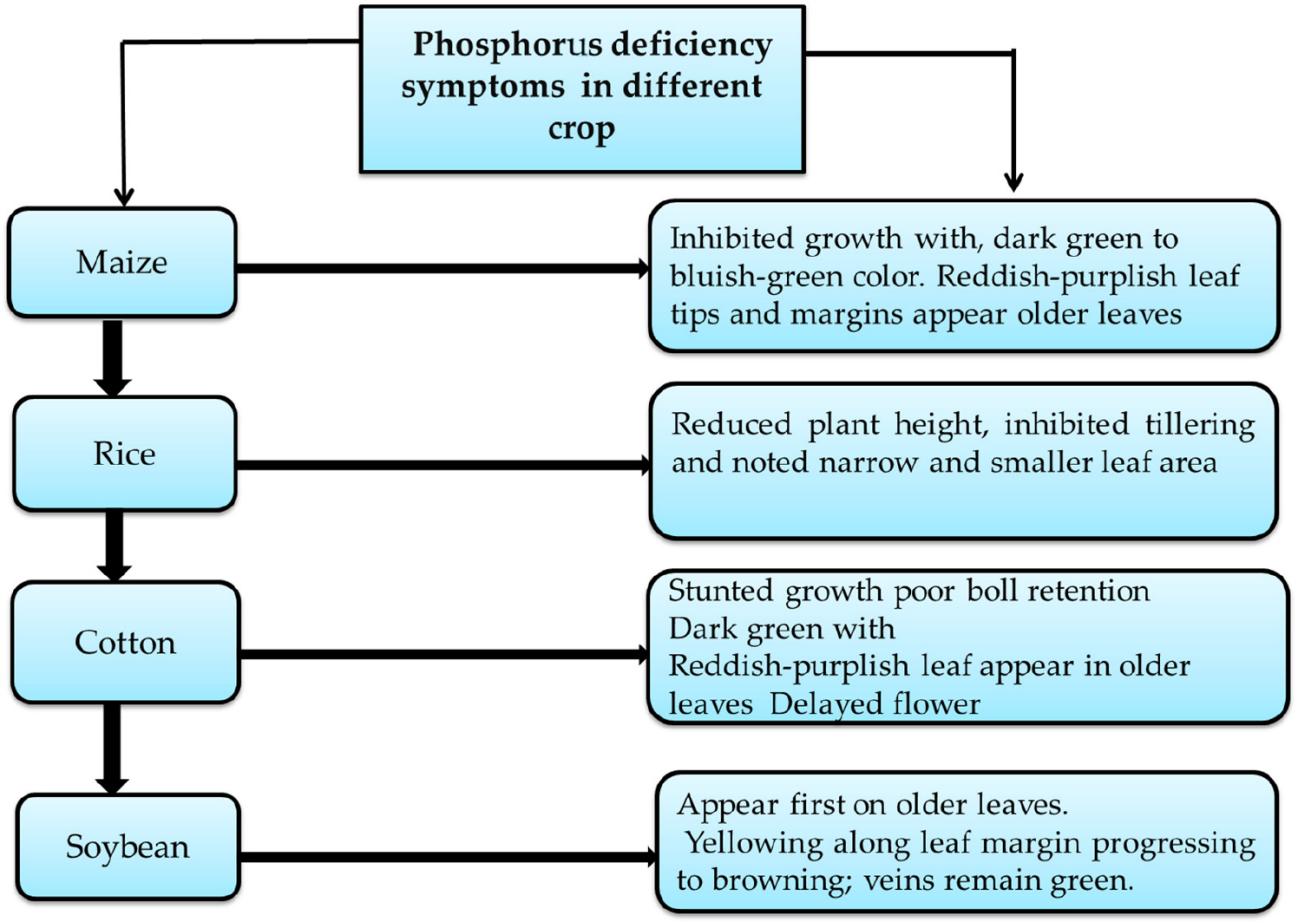
P deficiency symptom in different crops.

Appropriate diagnostics and farming practices are needed to enhance P absorption efficiency in plants. The plant-based diagnostic technique can be used as the basis of soil analysis to evaluate crop P requirements. Deficiency symptoms occur in plants if they cannot uptake any nutrients according to their specific needs. The P absorption amounts by species mostly depend on root traits, which differ among genotypes of several crops.^9^ Approximately 5.7 billion hectares of land worldwide have recorded low phosphorus supplies (0.1%) for the optimal production of several crops, including wheat, rice, cotton, and soybeans.^20^Figure 4 shows that deficiencies of P have adverse effects on crop production and their economic value. In most cases, P concentrations decrease with increasing above-ground biomass production. Research from Belanger et al.^27^ showed that rapeseed grain yield decreased due to P deficiency. In some cases, positive grain yield responses to P fertilization have been reported.^28^ It has been reported that phosphorus deficiency in plants is mostly due to inefficient chlorophyll production, resulting in leaf chlorosis. Symptoms first appear on older leaves; visual symptoms occur due to sudden abnormal anthocyanin levels, and the color changes to orange-red. These changes occur in leaf tips and then move to the base with necrosis or death of the leaf tip.^29^

Furthermore, continued P deficiency can increase the anthocyanin levels, subsequently followed by purple discoloration on the leaf surface.^30^ Plant P deficiency causes many problems with plant growth and yields, and some are described below. The deficiency of P in maize has produced adverse effects on production and quality, such as reducing morphological characteristics of roots and decreasing the capacity of water and nutrient uptake.^31^ In addition, Iqbal et al.^32^ reported that uncertain growth habit cotton exhibits morphological adaptation, such as modifying the canopy arrangement, with phosphorus (P) application. Thus, the phosphorus deficiency prevents cotton growth and, by decreasing the biomass accumulation, leads to a reduction in cotton seed production. The cotton cultivars suggested phosphorus availability can boost seed cotton production. Phosphorus deficiency leads to narrow and smaller leaf areas in rice plants. Extreme phosphorus deficiency had a greater effect on leaf elongation than equivalent potassium deficiency levels, but there was no difference between the effects of phosphorus and potassium deficiencies at moderate or mild phosphorus deficiency levels. In many plants, a prolonged reduction in the availability of P reduces their yield potential. However, plants have evolved to cope with low phosphorus availability, and they have developed mechanisms that can both increase phosphorus availability from the soil and improve its internal use.^33^ Previous literature demonstrated that a deficiency of phosphorus reduced the biomass of roots and shoots of lettuce and tomato and the decreased the leaf number of Chinese milk vetch, alfalfa, lettuce, tomato, and marigold plants.^34^ Phosphorus deficiency leads to reduced height, sharper leaf angles, inhibition of tillering, prolonged dormancy, premature senescence, and reduced size and number of flower and bud.^35^ P plays an important role in legume nodule formation and symbiotic nitrogen fixation. Several studies have shown that stimulation of strong nodules is associated with P.^36,37^ It is noted that under P deficiency legumes decrease their N_2_ fixation in the exchange for a greater preference for soil N uptake by roots.^38^ Plant tissue tests and the response of crops toward nutrient application are the most complete methods due to their expansiveness. Successful application of all the diagnostic techniques in correcting P deficiency in plants can increase profitability and minimize the environmental impact of fertilization. It is necessary to determine the plant P status and manage P fertilization for optimal plant nutrition.

## 3 P Loss by Soil Erosion and Its Management

In various landscape degradation processes, soil erosion is considered to be a major environmental problem, leading to the loss of topsoil and nutrients and reducing soil fertility.^39^ An excessive amount of legacy P loss is related to runoff from sloping land and can lead to degraded soil quality and further non-point-source pollution of downstream marine ecosystems.^40^ Runoff loss is affected by various factors such as rainfall, land distribution, and farming methods.^41^ An estimated 10 million hectares of farmland are abandoned worldwide each year, reducing productivity due to soil erosion.^42^

Loss of agricultural productivity, especially as a result of erosion, involves loss of income for socioeconomic development in developing countries whose economies are largely dependent on the agricultural sector.^43^ Globally, it is expected that the cost of land degradation is between 2.9% and 6.3% of the agricultural gross domestic product.^44^ Recent estimates of the cost of a degraded land show that the economic influence is highly uncertain, ranging from $4 billion to $490 billion; using macroeconomic modeling, the CGE recorded a cost of soil erosion to the European agricultural sector of approximately EUR 295 million per year (a 0.12% reduction), increasing to a GDP loss of approximately EUR 155 million.^45^ In the African continent, the annual loss of crop yield is about 280 million tons, compared to a consistent figure of only six million tons in the European Union.^46^ It is necessary to understand how soil losses occur directly, but there are different implements present to evaluate soil erosion from the landscape, climate, and controlling factors. Based on the Universal Soil Loss Equation (USLE), which is a mixed model that combines both source and transport factors to forecast soil transfer to the bottom of a 22.7 m slope.^47^ Applications of P fertilizers to the soil surface present a high amount of organic source of P, which can immediately runoff due to water.^48^

The interaction of fertilizers with soil and rainfall led to the dissolution of fertilizer particles over time as well as succeeding rainfall events. As a result, the solution is more concentrated compared with those produced by runoff water and soil interaction, so soil represents a good sink for desorbing phosphorus from fertilizers. In certain counties, most of the rainfall and later runoff occurs during the nongrowing season.^49^ In southern Europe, increased evapotranspiration reduced rainfall, and thus decreased water flow is also expected to lead to increased concentrations of nutrients, including phosphorus, especially in river basins dominated by wastewater treatment plant discharge or high background phosphorus losses.^50^ It is expected to decrease yearly runoff, very extreme precipitation events will be more frequent in Southern Europe and therefore erosion rates and phosphorus loads will increase.^51^ The one-season price of N, P, and K lost by erosion over a maize monocrop grown extremely tilled land was $7.1 per hectare.^52^ Manures are also an obtainable source of P for runoff but have two key differences. The first is that manure contains arrange of organic forms of P,^54^ and all are directly soluble, while some compounds will take time to release soluble P.^53^ The amount of release of these compounds can also differ with soil reactions, e.g., redox potential. The second is the physical form of the manure, as liquid manure may infiltrate immediately, reducing the effectiveness of incorporation due to the larger volume applied and the clumpy nature of the solid manure.^48^ However, some research is devoted to the economic impact of soil fertility, and the reduction of soil erosion under various management practices and sustainable cropping systems.^55^

Land cover with vegetation between the perennial crops is necessary to minimize soil erosion because about 50% of citrus orchards in Paraná State have been implanted in soil resulting from Caius sandstone, which has lower clay, little natural fertility, and potentially high water erosion.^56^ Perennial crop cultivation is the most attractive single practice for decreasing soil erosion because this method prevents the origin of the erosive process by decreasing the energy of raindrops impacting against the soil surface, thus avoiding the disaggregation of soil particles and facilitating higher water infiltration. Biochar-integrated practices in agricultural management systems promote properties of soil and crop production and protect the environment from pollution.^57^ The soil chemical characteristics are also affected by the addition of biochar in a more complex way, which helps reduce the erosion loss.^58^ Biochar application could maintain the soil pH in an acidic soil condition and absorb more cations.^59^ Major nutrient losses from maize cultivation were associated with decreased land cover with increased soil deposit transport. In contrast, the cowpea cropping system has good land covers, which have less of nutrient loss; it is emphasized that the planting of legumes plays a role in soil nutrient conservation. Therefore, the combined practice of chemical mineral fertilizers and biochar during cowpea cropping systems is expected to reduce profit losses from soil erosion in sub-Saharan African land.^60^ The accumulation of soil organic matter has been considered an effect of soil erosion from water and wind.^61^ Developed erosion surfaces can be controlled by maintaining and growing more vegetation surface area. However, there is also a need to reduce the impact of heavy rainfall and high-flow water used to control the application in land-use practices.

## 4 The Role of P Fertilizer and the Impact of P Deficiencies on Food Security

The increasing world population has led to increased agriculture production requirements.^62^ However, in this situation, the P fertilization technique should be applied to meet plant requirements Phosphorus is a vital element that can boost food production security on large scale in a sustainable agricultural ecosystem. Food security is related to agricultural farming practices and the availability of soil nutrients to support plant yield production. There is also a need to describe the effects of P deficiency on human food production. In most cases, fruit flowering and seed production of plants such as wheat, rice, and maize, which are important food crops around the world, are reduced when plants absorb enough phosphorus during their growing season. For example, it can easily reduce the protein content in grain crops. The P deficiency not only reduced the P concentration but also reduced the carotenoid content and total chlorophyll in tomato seedlings.^63^ However, P deficiency can change the metabolism and translocation of carbohydrates, such as soluble sugars and organic acids.^64^ Increased carbohydrate (especially sucrose) accumulation has been observed in the leaves of many plant species under the deficiency of phosphorus,^65^ which has been attributed to low sink requirements and limited leaf expansion during phosphorus deficiency. About 9.5 million tons of phosphorus enter human food, such as milk, fish, crops, and animals, and is then utilized; 3.5 Mt P is physically consumed by the human population, with the remaining 4.8 ± 1.3Mt/a of P processed as nonfood products (e.g., nonfood oils), wasted (e.g., spoiled food), or lost as inedible components (e.g., banana peels and egg shells) and predominantly destined for landfills or compost heaps.^66^ Simply a part of the use of limited resources is a buildup of legacy P, such as in US and European soils during P fertilizer applications, which also prevents environmental challenges.^67^ The consumption of different phosphate fertilizers has contributed to feeding billions of the world’s population over the last hundreds of years via improving crop production.^68^ It is predicted that the world population would increase to about 9 billion by 2050. It is necessary that at the same time agricultural strategies broadly improve food production by up to 80% to 90% by handling the global dilemma that is influenced by climatic changes. This is the main challenge related to the consumption of phosphorus to support future crop yield. Nearly 148 million metric tons of phosphate rock were used annually as phosphate fertilizers, and the source of phosphorus came from the mined phosphate rock, of which about 5 million metric tons of phosphate fertilizers are produced per year.^69^ The presence of P in the global food system typically begins in the mining industry, when phosphate rock is excavated, cleaned, and shifted to the fertilizer industry, where it is chemically processed into phosphate fertilizers. Phosphate fertilizers such as diammonium phosphate, monoammonium triple super phosphate, and single super phosphate are traded on a global scale, then enter the agricultural field and are regularly used in farmland and pastures.^70^ The main portion (80–90%) of chemical P fertilizers applied to the soil could be quickly immobilized, and the plant cannot easily uptake it due to its adsorption with iron (Fe-oxides/hydroxides), aluminum (Al-hydroxides), calcium (Ca^+^) carbonate, and Mg carbonate.

As a result, less than 20% of phosphorus fertilizer might be added to the soils, and typically a small amount of P fertilizer is taken up by plants.^5^ Chemical extraction could be used to analyze the soil P availability, which estimates the range of plant uptake and is a common recommendation for fertilizer application.^71^ P fertilizer application levels are high around the world, for example, 196 kg/ha in Asia and 5 kg/ha in sub-Saharan Africa.^72^ In Africa, P consumption is estimated at 2.8 t, which increased between 1975 and 2005.^73^ More than 50% of chemical fertilizers are applied in the Brazilian farming system.^74,75^ The consumption of P fertilizer has also been used to develop soil P levels in some areas, particularly in the western region of Europe.^76^ In China, wheat generally consumes the highest P input per unit area due to insufficient phosphorus from fertilizers and other sources, but it is less efficient than corn and rice.^77^ Continuously stopping the P fertilization for 14 years in winter in a wheat and maize growing field resulted in higher rate of Olsen P in the soil with the highest (27 mg kg^–1^) contents, and the lowest rate decreased in the soil with the lowest Olsen P concentration (4 mg kg^–1^).^78^ A quick reduction of the developed Olsen P content might be due to a higher proportion of P that is not easily available for uptake by plant roots. The overall application of inorganic phosphorus fertilizer on sugar cane in Brazil at planting is usually 50–80 kg P/ha, with an average of 35 kg P per hectare per year.^79^ Appropriate phosphate fertilizer application can improve crop quality; for example, band fertilization can improve the soil phosphate sequestration capacity more than the seeding method. This is because band applications, particularly mono- and diammonium phosphate fertilizer, develops the root system, which has an impact on P thereby improving the ability of plants to absorb phosphorus.

Current knowledge about phosphorus in soil and enhanced input techniques can help address this dilemma and stimulate more efficient use of phosphate fertilizers worldwide. Improving the efficiency of phosphate fertilizers could cover the life of P stocks, increase food requirements, and alleviate the environmental risks associated with excess phosphorus fertilizers often used.^80^

## 5 The Role of Green Manure Crops and Their Root Trait Mechanisms to Improve Soil P

It is necessary to assess crops and their adaptation to P stress conditions, and modern strategies can boost plant production can improve plant yields and decrease the reliance on costly artificial P fertilizers.^81^ The ability of plants to produce high yield quality in phosphorus-limited environments is called the plant’s P uptake efficiency.^82^ Improving the P absorption ability has long been an important challenge in complete cropping systems.^83^ The high P absorption capacities of plants depend on root characteristics such as root morphology, root architecture, greater root length density, root hair, root exudates, and association of mycorrhizae, which are are symbiotic associations based on nutrient transfer between soil, fungi and the vascular roots of plants.^18^ Green manure crops are well-known to increase soil fertility in the integrated and organic farming systems. Green manure crops including legumes and non-legume species not only improve soil quality and soil water content but also enhance nutrient availability via the N fixation capacity. The area of the plant’s roots in contact with the soil surface becomes a crucial factor, and root systems play essential roles in nutrient uptake. Green manure increased the soil microorganism population not only at the time of its incorporation but also the growth period as well, e.g., through root exudation, root turnover, and symbiosis with mycorrhiza.^84^ Leguminous plants are the basic factors that usually require greater P uptake levels in the soil, and legume root growth and root morphological mechanisms are involved in plant P uptake.^85^ Green manure crops such as *Lupines albas* L., *Cicer arietinum* L., *Pisum sativum* L, and *Vicia faba* are able to mobilize soil P through a variety of root mechanisms.^11^ Plant roots proliferate in this phosphorus-rich surface area, and a small fraction penetrates the subsoil, which stores most of the soil water that plants may use. For this reason, root distributions down to the soil profile and root length are significant for plants to take up. Plant roots can release several exudates, which contain monocarboxylic (e.g., lactic gluconic and lactic), dicarboxylic (e.g., oxalic, tartaric, malic, fumaric, and malonic) and triarboxylic acids (citrus); these different types of root exudates have an effect on P mobilization^86^

**Figure 5:**
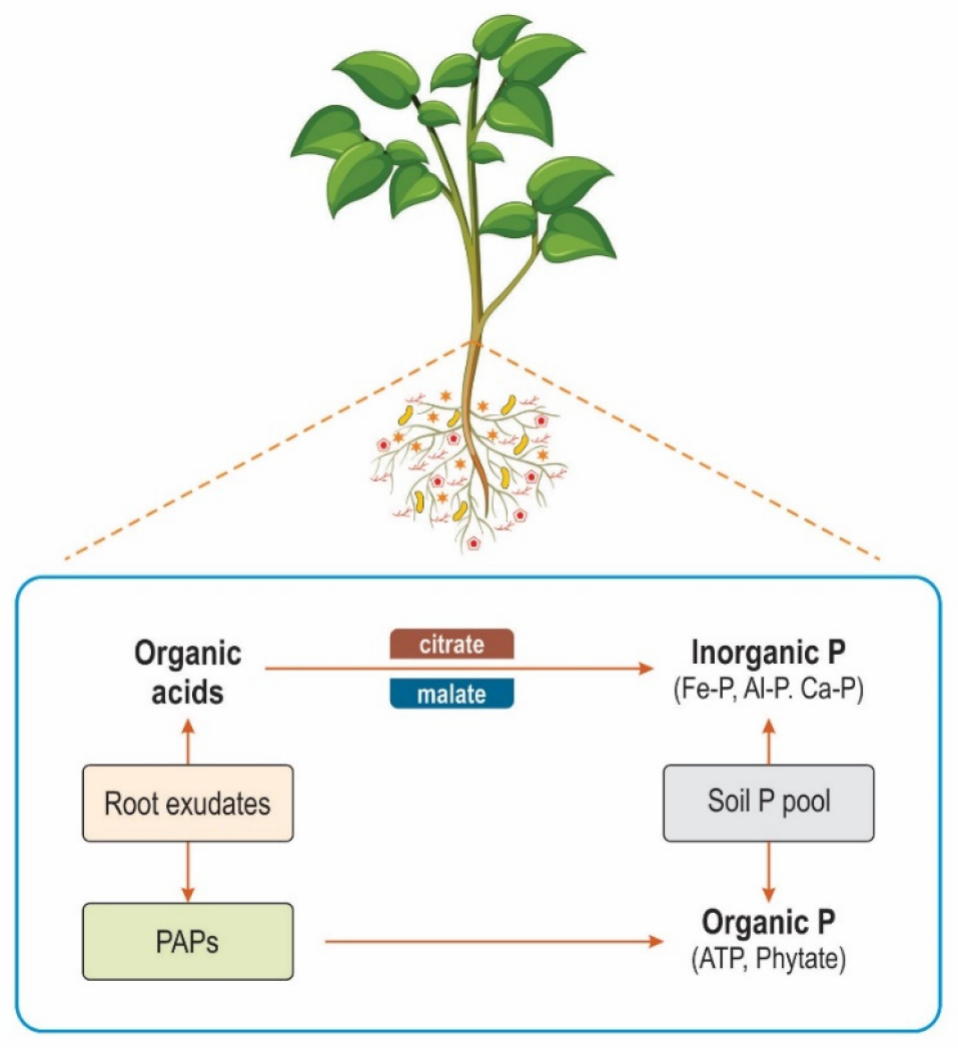
Organic and inorganic P forms.

The extensive root systems and the formation of root hair widely supply particular nutrients (e.g., NO^3–^ and P) to the plant. Roots hair enhances the ability of roots to explore the rhizosphere for P by increasing the surface area for absorption.^87^ Cultivars with improved P uptake ability could alternatives for overcoming the dilemma of P deficiencies. Green manure crops are unique due to their root mechanisms, while several varieties secrete the organic acids into the soil and improve the P solubilization, and plants can survive in low-P environmental conditions.^88^ However, Figure 5 shows the mechanisms of how roots release organic acids and organic and inorganic P forms. The root exudation process increases the plant growth, and the amount and pattern of root exudates might depend on different crop species. The thinner and cluster root formation of different varieties, such as common bean (*Phaseolus vulgaris* L.) and soybean, help scavenge the P uptake from topsoil. In addition, the secretion of organic compounds by leguminous plants, for example, chickpea, field pea, white lupin, and fava bean roots, is helpful for increasing the amount of soil-available P.^89^ The root-released citric acids from white lupin improve the P availability through solubilizing Al, Fe, and Ca phosphates.^11^ Moreover, wheat (*Triticum aestivum* L.), has special root traits (greater length density), which are essential for the plant to obtain P from top-soil layers.^90^ The synthesis and secretion of phosphatase by wheat roots have been observed to enhance P availability in the soil.^91^ Different rice genotypes with higher P absorption capacities can be utilized for the improvement of rice production.^92^ Enhanced internal P utilization efficiency is required to supplement higher P absorption traits for the successful breeding of P-efficient rice cultivars.^93^ In the phosphorus-efficient rice genotypes, greater P retransfer from old to young leaves is important to generate more photosynthesis, which ultimately increases biomass production. Maize genotypes with high P absorption abilities have the characteristics of vigorous root growth, large absorption area, strong root activity, and strong root affinity to P.

### 5.1 Role of Micro-Organisms in P Availability

Different organisms can regulate the P cycle, and mostly micro-organisms are helpful for P availability. The direct pathway for P absorption is through root hairs and root epidermis, confining P uptake to the soil volume closest to the root surface. However, plant-growth-promoting bacteria (PGPB) and arbuscular mycorrhizal fungi (AMF) both can attribute insoluble forms of soil P, such as inorganic and organic, throughout the world. Although linking mycorrhizae to root traits in terms of P acquisition has attracted a lot of attention,^94^ some micro-organisms have the ability to convert inorganic P into organic forms, also known as P-solubilized micro-organisms (PSM). Plants roots having a friendly beneficial interaction with PSM, PGPB, and AMF could increase the P uptake in the plant.^95^ When plant species potentially release organic acid (H^+^ hydron and hydroxide OH^–^), phosphate activity helps to improve the amount of available inorganic and organic P and contributes to plant P nutrition.^96^

The use of phosphate-dissolving bacteria (PSB), such as *Azotobacter*, *Bacillus*, *Micrococcus*, *Pantoea agglomerans*, *Achromobacter*, *Aereobacter*, *Arthrobacter*, *Pantoea agglomerans*, *Aspergillus*, *Bradyrhizobium*, *Burkholderia*, *Agrobacterium*, *Alcaligenes*, *Chromobacterium*, *Enterobacter*, *Escherichia*, *Flavobacterium*, *Klebsiella*, *Micrococcus*, *Pseudomonas*, *Rhizobium*, and *Salmonella*, could represent a management strategy for improving P use efficiency in plants. Attention has been given to the rhizosphere and plant–soil-based interface at the root hair. The phosphate-solubilizing fungi (PSF), including *Alternaria*, *Arbuscular mycorrhiza*, *Aspergillus*, *Fusarium*, *Helminthosparium*, *Penicillium*, and *Rhizopus*, are related to plant root hair and accumulate approximately 1–50% of the whole amount of P in the soil, with phosphate-dissolving actinomycetes (PSA) playing an additional secondary role, i.e., *Streptomyces* and *Nocardia*.^97^

Different bacteria significantly have different abilities to dissolve phosphorus in the soil status. Bacteria that are identified to increase P availability include species of *Pseudomonas*, *Azotobacter*, *Burkholderia*, *Bacillus*, and Rhizobium.^98^ Mycorrhizal symbioses contribute significantly to plant nutrition, particularly to phosphorus uptake. Phosphate-dissolving fungi could develop more acids compared to bacteria and therefore show higher P solubilizing activity. Filamentous fungi identified to be able to solubilize phosphate include the genera *Aspergillus* and *Penicillium*.^99^ In some cases, mycorrhizal fungi interacting with microorganisms release enzymes that can break down the unsoluble P compound.^100^ Because the mycorrhizal fungi have a very large surface area, their hyphae network is efficient for nutrient uptake, mainly for phosphorus.^101^ It is noted that inoculation of AMF reduced the recommended phosphate fertilizer rate about 80%.^102^ The P-solubilizing ability of actinomycetes has attracted interest because these groups of soil organisms are not only able to survive in drought conditions but also potentially produce phytohormone and antibiotics compounds that could simultaneously be helpful for crop production.^103^ The P solubilization capacity different micro-organisms is mainly dependent on plant varieties and soil fertility status. Some important phosphorus cycle enzymes, such as phosphatases, phosphohydrolases, phytases, C–P lyases, and phosphatases, are strongly secreted by microorganisms that catalyze the mineralization of organophosphates. Several quantities of extra-cellular and intracellular enzymes are present in the soil.

Phosphatase is the most commonly secreted enzyme that reduces P from its substrate by hydrolyzing phosphoric acid monoesters into a P ion and a molecule with a free hydroxyl group and phytase because of the major existence of their substrates in soil.^104^ A large class of enzymes containing phosphatases and phosphohydrolases catalyze the hydrolysis of esters and anhydrides of H_3_PO_4_.^98^ Phytase (myoinositol hexaphosphate phosphohydrolase) hydrolyze sodium phytate and, as a result, increased the amount of inorganic P. The phosphate ions immediately bound with other cations and formed insoluble complexes in the soil, which plants are not able to absorb,^105^ and the occurrence of mycorrhizal fungi (MF) is helpful for P solubilization. The activities of soil enzymes play a crucial role in the degradation of complex molecules into simple ones as well as the improvement of soil fertility levels.^106^ They potentially provide a unique biological assessment of soils due to their interaction with microorganisms and their quick responses to modify soil conditions. Extracellular phosphate enzymes have been typically linked with the transformation of soil P, as they catalyze the hydrolysis of ester–phosphate bonds, which leads to the release of the phosphate and allows plants to survive in P stress conditions. These phosphatase enzymes are released by several bacteria, fungi, and plant roots. Acid phosphate activity is mostly higher in the upper layer of soil (humus) and decreases with increasing soil depth. The soil phosphate activity depends on many factors, including soil properties, relationship with soil microbes, and the presence of inhibitors and activators. Hence, soil pH (acidic 3–5.5 and alkaline 8.5–11.5) is essential for the proper functioning of the soil enzymes.^107^ The phosphatase enzyme necessary for the P cycle requires both pH ranges. The roots of different cereal crops such as wheat, sorghum, and millet and three different leguminous, i.e., cluster bean, mung bean, and moth bean, exhibited activities of acid phosphates when these species were mainly grown in cultural solution under phosphorus stress conditions. As phosphates decrease the the soil status, the enzymes released via plants may mineralize the organic phosphorus into soil-available phosphorus.

### 5.2 Agricultural Practices

On the other hand, many studies have focused on crop rotation and intercropping practices, which are mainly used to develop the soil P content.^108^ Crop rotation could promote greater P use throughout the rotation.^109^ Green manure rotation improves the soil nutrient supply capacity, improves the quality and quantity of soil organic matter, has a positive impact on soil nutrient availability, and contributes to the sustainability of rice yields.^110^ Wheat is alternated with fallow in some temperate dry land environments as a winter crop, with summer fallow in subtropical environments, and in Australia in a phased rotation with pastures, where wheat acreage is declining, being dominated by annual legumes (currently about 7 million hectares).^111^ Root interactions in mixed cropping systems are most likely of importance for the nutritional improvement of crops grown in low-nutrient-condition agro-ecosystems.^112^ Intercropping cowpea–maize led to an increase in P availability at the rhizosphere level that was associated with significant acidification compared to that in single cropping.^113^ An earlier study demonstrated that legumes are valuable in improving the N and P nutrition of pearl millet, and chickpea facilitated P uptake by maize and wheat.^114^ Intercropping wheat and chickpea increases biomass yield by 4.4% and 3.5% in drought condition as compared to monocrop wheat and monocrop chickpea, respectively.^115^ Measuring properties related to N and P cycling in the rhizosphere of wheat and leguminous varieties, i.e., white lupine and fava bean grown in monoculture (wheat/legume mixtures), showed that the less-labile organic P pools significantly collected in the rhizosphere of legumes. However, the changes in rhizosphere soil P pools and acquired P depend on legume species. Different legumes like lentils, chickpeas, peas, and fava beans play a major role in protection agriculture in North America, Australia, and Turkey. The European scarcity in leguminous plants is not reflected in other areas of the world such as Canada or Australia, where legumes have been increasing over the last few decades.^116^ The highest production of wheat is observed after legume cultivation, including chickpeas, fava beans, lentils, and field peas, compared to the production of wheat after wheat in Australia.^117^ Earlier studies showed, particularly for wheat, that intercropping with wheat could produce 4.0 t/ha, and the average mean of legume intercropping with wheat led to 5.2 t/ha (30%) higher production. Cultivation of pastures legumes like *Lucerne* sp. to fix nitrogen for next crop rotation of maize and wheat-soybean and Olsen P content were reduced by half as compared to only growing grain crops. Forage grasses and grain rotations were able to decrease the amount of topsoil P 11–36% over 13 years in Swedish lysimeter trials.

## 8 Conclusion

Phosphorus plays a vital role in plant metabolic activities, which are used in human food production. Phosphorus deficiency reduces crop production, which has an adverse effect on human food products and reduces food mineral levels and nutritional value. The wheat rice and maize are important food crops throughout the world and can easily exhibit reduced fruit, flower, and seed production, and also reduced protein content in grain, when grown in P insufficient environmental conditions. While P deficiency decreases the carotenoid content and total chlorophyll in tomato seedlings, it also can change the metabolism and translocation of carbohydrates, such as soluble sugars and organic acids. Increased accumulation of carbohydrates, especially sucrose, has been observed in the leaves of many plant species deficient in phosphorus. Despite P being abundant in soil, 40% of the agricultural soils are deficient in phosphorus due to immobilization and slow uptake of phosphorus ions from soil to roots. Throughout the world, pproximately 10 million hectares of fertile soil exhibit reduced productivity due to soil erosion. Intercropping practices improve the production of wheat; when intercropping occurs between wheat and chickpea, it also improves the mineral nutrition of wheat and chickpea seed. Different agricultural techniques including intercropping, crop rotation, and legume-based cropping systems decreased soil nutrient loss with te lowest economic cost. These farming practices reduce the cost of chemical fertilization and are being used worldwide to improve P-deficient soils. However, it is noted that continuously stopping P fertilization application declines the available P content of the soil. Further knowledge needs to clarify, assess, and identify leaf appearances for different stages of plant growth, which would contribute to improving the P diagnostic effect.
